# Whole-genome sequencing reveals clonal expansion of multiresistant *Staphylococcus haemolyticus* in European hospitals

**DOI:** 10.1093/jac/dku271

**Published:** 2014-07-17

**Authors:** Jorunn Pauline Cavanagh, Erik Hjerde, Matthew T. G. Holden, Tim Kahlke, Claus Klingenberg, Trond Flægstad, Julian Parkhill, Stephen D. Bentley, Johanna U. Ericson Sollid

**Affiliations:** 1 Department of Paediatrics, University Hospital of North Norway, Tromsø, Norway; 2 Department of Clinical Medicine, UiT The Arctic University of Norway, Tromsø, Norway; 3 Department of Chemistry, Norstruct, UiT The Arctic University of Norway, Tromsø, Norway; 4 The Wellcome Trust Sanger Institute, Hinxton, Cambridge, UK; 5 Department of Medicine, University of Cambridge, Addenbrookes Hospital, Hills Road, Cambridge CB2 0SP, UK; 6 Department of Medical Biology, UiT The Arctic University of Norway, Tromsø, Norway

**Keywords:** staphylococci, SCC*mec*, molecular epidemiology, multidrug resistance, bacterial genomics

## Abstract

**Objectives:**

*Staphylococcus haemolyticus* is an emerging cause of nosocomial infections, primarily affecting immunocompromised patients. A comparative genomic analysis was performed on clinical *S. haemolyticus* isolates to investigate their genetic relationship and explore the coding sequences with respect to antimicrobial resistance determinants and putative hospital adaptation.

**Methods:**

Whole-genome sequencing was performed on 134 isolates of *S. haemolyticus* from geographically diverse origins (Belgium, 2; Germany, 10; Japan, 13; Norway, 54; Spain, 2; Switzerland, 43; UK, 9; USA, 1). Each genome was individually assembled. Protein coding sequences (CDSs) were predicted and homologous genes were categorized into three types: Type I, core genes, homologues present in all strains; Type II, unique core genes, homologues shared by only a subgroup of strains; and Type III, unique genes, strain-specific CDSs. The phylogenetic relationship between the isolates was built from variable sites in the form of single nucleotide polymorphisms (SNPs) in the core genome and used to construct a maximum likelihood phylogeny.

**Results:**

SNPs in the genome core regions divided the isolates into one major group of 126 isolates and one minor group of isolates with highly diverse genomes. The major group was further subdivided into seven clades (A–G), of which four (A–D) encompassed isolates only from Europe. Antimicrobial multiresistance was observed in 77.7% of the collection. High levels of homologous recombination were detected in genes involved in adherence, staphylococcal host adaptation and bacterial cell communication.

**Conclusions:**

The presence of several successful and highly resistant clones underlines the adaptive potential of this opportunistic pathogen.

## Introduction

*Staphylococcus haemolyticus* is an emerging cause of nosocomial infections, in particular affecting very preterm infants and immunosuppressed patients.^1,2^ Among the coagulase-negative staphylococci (CoNS), *S. haemolyticus* is now the second most frequently isolated species from human blood cultures, after *Staphylococcus epidermidis. S. haemolyticus* is often resistant to commonly used antimicrobial agents and is ranked as the most antibiotic-resistant CoNS species.^3^ The development and spread of antimicrobial resistance present a global threat to modern medicine by limiting the available treatment options. Mobile genetic elements (MGEs) including plasmids, prophages, transposons and pathogenicity islands, conferring resistance to commonly encountered antimicrobial agents, enable rapid adaption to changing environments.^4^ The sequence similarity of MGEs and resistance genes in different *Staphylococcus* species suggests that these genetic elements are readily transferred between staphylococci.^5^ It has been hypothesized that in *Staphylococcus aureus* both the methicillin resistance gene *mecA*, carried on the staphylococcal cassette chromosome (SCC*mec*), and the arginine catabolic mobile element (ACME) originate from CoNS.^6^ This suggests that CoNS play an important role in the dissemination of resistance genes,^7^ contributing to the emergence of epidemic clones of the more virulent nosocomial pathogen *S. aureus*.

Population studies of *S. aureus* have revealed the establishment of several successful clones.^8^ Whole-genome sequencing (WGS) has offered high-resolution insight into their emergence, expansion and transmission.^9^ Persistent multiresistant hospital-adapted clones have also been detected in *S. epidermidis*.^10^ The presence of endemic *S. haemolyticus* hospital clones and the nosocomial spread of resistant clones have been reported.^1^ However, little is known about the population structure and the possible emergence of hospital-adapted clones and factors contributing to the persistence of *S. haemolyticus* in the hospital environment. Previous molecular epidemiological analyses performed by multilocus sequence typing (MLST) and multilocus variable number of tandem repeats (MLVA) analysis did not confer satisfactory discriminatory capacity for a detailed molecular epidemiological analysis.^11^ Furthermore, comparative genome studies of *S. haemolyticus* have to date been limited by the presence of only one sequenced *S. haemolyticus* isolate (JCSC 1435).^12^

We performed WGS of 134 diverse geographical and temporal clinical *S. haemolyticus* isolates. In order to understand the molecular basis of the emergence of *S. haemolyticus* as a nosocomial pathogen, we performed comparative genomic analysis of the 134 isolates to investigate their genetic relationship and explore their evolutionary history regarding the development of antimicrobial resistance and adaptation. The presence of host restriction and modification (RM) systems, which are known to limit the interspecies and intraspecies uptake of foreign DNA,^13^ was investigated in order to understand the diversity of the MGEs causing the previously high levels of antimicrobial resistance observed in *S. haemolyticus*.

## Materials and methods

### Bacterial isolates

A total of 134 isolates of *S. haemolyticus* from geographically diverse origins (Belgium, 2; Germany, 10; Japan, 13; Norway, 54; Spain, 2; Switzerland, 43; UK, 9; and USA, 1) were sequenced (Table S1, available as Supplementary data at *JAC* Online). The majority, apart from five community isolates, were blood culture isolates from catheters or wounds, isolated within the period from 1988–2010. The Swiss isolates were collected between 2001 and 2008, the Norwegian isolates were collected between 1988 and 2005, the UK isolates were collected in 2005 and the German and Spanish isolates were collected in 2008, the most recently collected isolates were from Belgium, collected in 2010. Four isolates were of animal origin (bovine, equine and porcine). The collection represented clinical isolates from eight countries, isolated over a 22 year period. Two large groups of isolates originating from one Swiss (*n* = 43) and two Norwegian hospitals (*n* = 54) were chosen for an investigation of clonal establishment within a geographically confined hospital setting. The inclusion of 18 blood culture isolates from a single neonatal unit in one of the Norwegian hospitals allowed us to investigate local adaptation. For the sequenced clinical isolates, no pre-selection based on antimicrobial resistance patterns was performed. Forty-one out of 45 isolates previously typed by MLST and MLVA were included for WGS.^11^ Overall the experimental design allowed an insight with differential resolution levels into the emergence of *S. haemolyticus* by investigating the establishment of international clones and the development of clones within single wards in single hospitals.

Genomic DNA was isolated according to Chachaty and Saulnier,^14^ with the addition of RNase A (10 mg/mL; Qiagen).

Susceptibility to antimicrobial agents (gentamicin, erythromycin, oxacillin, fusidic acid, tetracycline, ciprofloxacin, rifampicin and vancomycin) was performed for all isolates by Etest according to the manufacturer's description (AB BIODISK, Sweden). Additional susceptibility testing for trimethoprim, mupirocin, amikacin, linezolid, fosfomycin and daptomycin was performed on a number on isolates. Phenotypic resistance was interpreted by antimicrobial breakpoints according to the EUCAST guidelines.^15^ Multidrug resistance was reported as resistance to more than three unique classes of antimicrobial agents.

### Genome sequencing and annotation

WGS was performed on index-tagged libraries (76 bp reads with an average insert size of 400 bp) for each *S. haemolyticus* strain using an Illumina Genome Analyzer GAII as described in Bentley *et al*.^16^ Data have been deposited in the European Nucleotide Archive under the study number ERP000943. Each genome was assembled individually using Velvet^17^ and contigs >500 bp were ordered relative to the complete reference genome sequence of *S. haemolyticus* JCSC 1435 (AP006716) using ABACAS.^18^ Contigs were separated by the spacer sequence 5′-CTAGCTAGCTAG-3′ introducing stop codons in all six reading frames. Protein coding sequences (CDSs) were predicted using Glimmer 3.^19^ Functional annotations were transferred from the fully sequenced *S. haemolyticus* JCSC 1435. The unique regions were annotated using an in-house genome annotation pipeline (GePan), combining best functional hits from BLASTp,^20^ Pfam^21^ and Priam.^22^

### Clustering of homologues

Clustering of homologous genes was performed using OrthoMCL^23^ on the CDSs from all 134 *S. haemolyticus* isolates. In addition, the CDSs of the fully sequenced *S. haemolyticus* JCSC 1435 were included in the analysis. Due to the genome homogeny, a relatively conservative parameter configuration for clustering was chosen. The BLAST^20^ percent identity cut-off was set to 70% and the OrthoMCL inflation parameter was set to 7. Unique identifiers were assigned to each isolate and all CDSs predicted within each isolate in the dataset. From this, each cluster of homologues was then represented by the combination of isolates and the genes that were included in it. The CDSs in the clusters were divided into three types: Type I, core genes, homologues present in all strains; Type II, unique core genes, homologues shared by only a subgroup of strains; and Type III, accessory genes, strain-specific CDSs.

### Phylogenetic analysis

Sequence reads from each strain were mapped onto the concatenated reference genome of *S. haemolyticus* strain JCSC 1435 (consisting of the chromosome and three plasmids, pSHaeA, pSHaeB and pSHaeC; accession numbers: AP006716, AP006717, AP0067178 and AP0067179) using SSAHA v2.2.1.^24^ Single nucleotide polymorphisms (SNPs) were identified as described in Croucher *et al*.^25^ SNPs in regions predicted to have arisen by homologous recombination were identified using Gubbins^25^ as previously described and were excluded from the phylogenetic reconstruction. The phylogenetic relationship between the isolates was built using RAxML v0.7.4 from variable sites in the form of SNPs in the core genome and used to construct a maximum likelihood phylogeny. SNPs in non-core regions of the genome were omitted from this analysis since the non-core regions are more susceptible to the horizontal acquisition of foreign DNA and could confound the interpretation of the tree.

### Host restriction systems

*S. haemolyticus* homologues of all RM enzymes in the REBASE^26^ were identified using BLASTp^20^ with an E-value cut-off <1e-5. The alignments of all potential matches were manually inspected to ensure that the sequences were of similar length. Due to the fragmented nature of the assembled genomes, the exact number of complete host restriction systems was not determined.

## Results and discussion

### Genome composition

The *de novo* assembly generated an average of 275 contigs per isolate. A total of 334 578 CDSs were identified in 135 isolates, including the reference strain JCSC 1435.^12^ OrthoMCL was used to cluster homologues based on sequence similarity and allowed us to group the genes into core genes that were shared among all isolates, accessory genes shared between some but not all isolates, and isolate-specific genes. All together we identified 5088 clusters, of which 1868 clusters contained core genes, 3187 were accessory gene clusters and 33 were isolate-specific clusters. For each isolate, around 25.1% of the total CDSs were accessory genes. These genes had either been acquired by horizontal gene transfer (HGT) in some isolates or excised from the genetic repertoire of others. Functional annotation of the accessory genes and mapping against staphylococcal MGEs showed that around half of the accessory genes were associated with MGEs. MGEs were identified and annotated by comparing all the unique and accessory genes against a database consisting of staphylococcal plasmids and prophages from the European Bioinformatics Institute^27^ and *S. aureus* pathogenicity island, transposons and SCC*mec* sequences as described by Holden *et al.*^28^ The remaining accessory genes had functions involved in metabolism, cellular processes and signalling. Some of these genes may also reside on novel MGEs not present in the reference set.

### Phylogenetic reconstruction based on core genes

In order to discern the establishment of clones, a phylogenetic reconstruction based on SNPs in the core regions of the genomes was performed. This separated the 134 isolates, excluding the JCSC 1435 strain, into one major group of 126 isolates and one minor group comprising eight isolates. The average diversity compared with the reference strain JCSC 1435 was 10 495 SNPs per genome in the major group and 64 428 SNPs per genome in the minor group. To refine the phylogeny, the eight genetically divergent isolates were omitted from further analyses.

**Figure 1.**
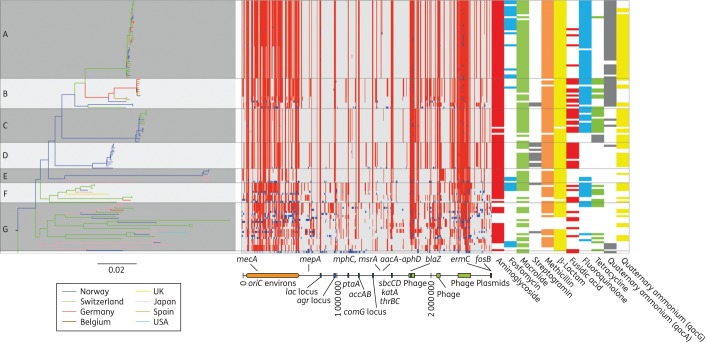
Phylogeography, recombination map and antibiotic resistance distribution of *S. haemolyticus*. Left panel: a maximum likelihood tree constructed from substitutions in the core genome of *S. haemolyticus*. The branch length of the outgroup has been shortened by a factor of 100 for better resolution of the tree. Branches are coloured according to geographical location. Middle panel: the chromosomal location of recombinations detected in the collection of isolates relative to JCSC 1435. Red blocks are recombinations predicted to have occurred on an internal branch and are shared by multiple isolates through common descent. Blue blocks are recombinations predicted to have occurred on terminal branches and are present in only one strain. The annotations of highly variable areas are shown below. In the collection of isolates sequenced, variation predicted to be due to recombination was identified in regions mapping to 78.1% of the JCSC1435 chromosome. Right panel: distribution of antibiotic resistance. Coloured slots represent identified genotypes corresponding with resistance to the antibiotic class indicated below. This figure appears in colour in the online version of *JAC* and in black and white in the print version of *JAC*.

The major group of 126 isolates was subdivided into seven clades (A–G). Clades A–D encompassed isolates from Europe only (Figure 1), reflecting the fact that the majority of isolates (93/126; 73.8%) originated from two hospitals in Norway and one in Switzerland. Clade D was only found in neonatal blood culture isolates from a single Norwegian neonatal ward and isolated during the period 1991 to 2003, 9 of the 13 isolates were isolated between 1991 and 1999. Isolates originating from Japan were found in Clades E–G. The increased resolution of WGS relative to MLST allowed the separation of isolates belonging to sequence type (ST) 1, into the two distinct Clades A and B (ST1-A and ST1-B). On the other hand, the congruence between WGS and PFGE was low. This possibly reflects the variation conferred by the accessory genes observed by PFGE, as opposed to the variation in the core genome used to construct the phylogenetic relationship by WGS.^11^ Clade C was composed of Norwegian and Swiss isolates belonging to ST4 and ST13, while the majority of isolates in Clade D were previously defined to be of the same (PFGE) type.^29^ Interestingly, the Norwegian isolates in Clades A, B and D originated from the same Norwegian hospital in South Norway, while isolates from the second Norwegian hospital in North Norway were mainly found in Clades C and E. In addition to isolates from Norway, Clade E also consisted of closely related *S. haemolyticus* isolates from Japan. The two remaining clades (F and G) accounted for 25.6% of the major group and were composed of geographically and temporally diverse isolates with a larger degree of variation as visualized by the longer branch lengths in Figure 1. Seven of the isolates from Clades F and G were previously grouped by MLST as belonging to clonal complex (CC) 2.^11^ This apparent global distribution of clones not necessarily following national borders might reflect international travel and the subsequent introduction of novel successful strains.^28^

The extensive sampling from a single neonatal ward formed the basis for a detailed study of a geographically constricted group of isolates. Thirteen out of 18 isolates from this ward, isolated in the period from 1991–2003 (9 of the 13 isolates were isolated between 1991 and 1999), were found in Clade D. The phylogenetic analysis suggests that this is a successful clone that has thrived in this particular environment. The remaining five isolates from this ward were found in Clade A and had been isolated from 2004 to 2005, indicating that there has been a replacement of the existing endemic clone at the neonatal ward from 2003 onwards. A global dissemination of successful clones, replacing established clones, due to human travel and activity has been demonstrated for several staphylococci including *S. aureus* and members of the CoNS such as *S. epidermidis* and *Staphylococcus hominis*.^28,30,31^ The ability of *S. haemolyticus* to disseminate and establish successful clones within European hospitals is clearly visualized through the relatively conserved yet geographically and temporally diverse composition of isolates in Clade A.

### Genome flexibility

The acquisition and loss of genes has shaped the genome of *S. haemolyticus*, but the genetic diversity is also the result of extensive recombination events. Site-specific recombination generally results in the integration or excision of DNA and can introduce MGEs into the genome, while homologous recombination involves the reciprocal exchange of identical or very similar DNA.^32^ Frequent homologous recombination was detected in genes involved in adherence, staphylococcal host adaptation and bacterial cell communication (Figure 1). This includes genes such as *sraP*, encoding the surface bound protein serine-rich adhesin for platelets, which is involved in bacterial aggregation in *S. haemolyticus*,^12^ and the mannitol-specific phosphotransferase genes (*mtlD* and *mtlA*). Evidence of frequent recombination in *sraP* was found as the pattern of variation in isolates belonging to Clades A–F showed individual recombinatory patterns. *mtlD* and *mtlA*, important in distinguishing *S. aureus*, *Staphylococcus saprophyticus* and *S. haemolyticus* from other staphylococcal species, were detected in 90 isolates.^12^ In *S. aureus* mannitol metabolism is important in response to antimicrobial fatty acids secreted on the skin and in abscesses by the innate immune system. *S. aureus mtlD* mutants displayed reduced growth in the presence of long chain fatty acids and increased susceptibility to H_2_O_2_.^33^ From the high prevalence in our collection (90/126), it is likely that the *mtl* operon is equally important for survival and host adaption in *S. haemolyticus*. Both *sraP* and the *mtl* genes are located in the *oriC* environs and the *mtl* genes have previously been reported to be easily lost in *S. haemolyticus*, indicating horizontal acquisition.^12^ *sraP* is flanked by transposon ISSha*1*, possibly contributing to mobilization.

The distribution of SNPs in the isolates in Clades A–G relative to the JCSC 1435 reference genome displays conserved regions, interspersed with highly variable regions throughout the genome (Figure 1). In particular, the region designated the *oriC* environs, which among others harbours SCC*mec*, is highly variable in our isolates. The high number of different recombinases (*n* = 6) and SCC elements (Table 1) may act as mediators for the frequent recombination events. The *oriC* environs has been suggested to confer a diversifying power separating the different staphylococcal species, enabling adaption to survival within the human host by acting as a hotspot of recombination.^12^ Our analyses show that differential recombinations within the *oriC* environs contribute to an increased diversity in *S. haemolyticus*, both within and between different clades. *S. aureus* is dominated by a few major clones in which recombination occurs but on a smaller scale.^8,34^ Recombination resulting in positive selection has been shown to function as an evolutionary driving force in other bacterial genomes.^35,36^ It is possible that the overall high recombination frequency in *S. haemolyticus* reflects an ongoing adaption to a recent change in lifestyle or selective pressure associated with the rather recent emergence of *S. haemolyticus* as an opportunistic pathogen in the hospital niche primarily associated with immunocompromised patients.

**Table 1.** Type and prevalence of recombinases and *mec* gene complexes

*ccr* gene complex^47^	*mec* gene complex^47^	No of isolates
AB1	class C	3
AB3	class C	4
AB1+AB_SHP_	class C	4
AB1 + AB_SHP_ + C	class C	1
AB2	class C	2
AB2 + AB4	class A	1
AB2, AB4, AB_SHP_, CcrC	class A	2
AB3	class C	4
AB3 + AB_SHP_	class C	1
AB3 + C	class C	1
AB4	class C	37
AB4 + C	class C	5
AB4 + AB_SHP_	class C	2
C	class C	25
No recombinases	class C	15
C	Δ *mecA*	3
AB4	Δ *mecA*	3
AB1	Δ *mecA*	3
AB1 + AB4	Δ *mecA*	1
A1B1 + AB3	Δ *mecA*	1
AB4 + AB_SHP_	Δ *mecA*	1
AB4 + C	Δ *mecA*	3

### Host RM systems

It has been demonstrated that the Type I and III RM systems prevent HGT by the recognition and removal of exogenous unmethylated DNA, whereas the Type IV RM cleaves DNA upon the recognition of modified cysteine residues.^13^ In our strain collection, Type I, Type II, Type III and Type IV RM systems were detected and several isolates harboured more than one RM system (Table S2, available as Supplementary data at *JAC* Online). Fifteen isolates harboured a functional RM system homologous to Sau1 Type I,^37^ 15 isolates had a Type II RM system, homologous to Sau3AI^38^ and 55 isolates had a Type IV system homologous to SauUSI.^39^ These RM systems have been described in *S. aureus.* As they may provide barriers to the exchange of genetic material, the number of RM systems is surprisingly high relative to the large presence of MGEs. However, within distinct RM groups there could be extensive genetic exchange. Recent studies have pointed to additional functions of RM systems, such as bacterial cell death as a result of RM systems acting as selfish DNA, maintaining their own existence in bacterial genomes.^40^ Additionally, RM systems are often co-localized on MGEs, possibly stabilizing the maintenance of these genes in the host.^40^

### Antimicrobial resistance determinants

*S. haemolyticus* has previously been reported to be multiresistant,^3^ and in this study multiresistance (i.e. resistance to at least three antimicrobial classes) was observed in 105/135 (77.7%) isolates (Table 2). The clade-wise prevalence of different antimicrobial resistance genes is shown in Figure 1 and the genes conferring resistance are listed in Table S1. Resistance to linezolid and vancomycin was not detected. Phenotypic observations of resistance correlated well with the cognate resistance genes. Discrepancy values ranged from 0.7% to 3.7% for aminoglycosides, macrolides, β-lactams (*blaZ* and *mecA*), fluoroquinolones, rifampicin, quaternary ammonium compounds and trimethoprim.

**Table 2.** Prevalence of antimicrobial resistance in the sequenced *S. haemolyticus* collection according to geographical origin

Country	Number	Presence of genes and mutations conferring antimicrobial resistance (%)									
		GEN	ERY	OXA	FUS	TET	CIP	RIF	TMP	QAC	FOF
Belgium	2	100	100	100	100	100	100	0	100	100	0
Germany	10	90	90	90	40	10	90	20	40	90	50
Japan	14	64	64	100	0	43	57	0	0	21	46
Norway	54	83	74	83	35	30	41	0	11	69	7
Spain	2	100	50	100	50	50	100	0	100	50	0
Switzerland	43	79	90	70	42	28	63	2	12	79	33
UK	9	78	78	78	44	67	67	22	56	78	22
USA	1	100	0	100	0	0	0	0	0	0	0

GEN, gentamicin; ERY, erythromycin; OXA, oxacillin; FUS, fusidic acid; TET, tetracycline; CIP, ciprofloxacin; RIF, rifampicin; TMP, trimethoprim; QAC, quaternary ammonium compounds; FOF, fosfomycin.

The horizontal transfer of resistance genes located on plasmids was apparent by variability not only between, but also within the clades. Plasmids encoding resistance to fosfomycin (*fosB*), fusidic acid (*fusB*), tetracycline [*tet*(L) or *tet*(M)], quaternary ammonium compounds (*qacA* or *qacG*), macrolides–lincosamides [*erm*(C), *erm*(A) or *vga*(A)] and trimethoprim (*dfrD*) were not uniformly distributed within the clades. Clades A and B were differentiated by the carriage of *erm*(C) and *tet*(L), respectively, whereas in Clade D no plasmids carrying *erm*(C), *tet*(L) or *fosB* were found. Plasmids carrying *fosB* were found in Swiss, German and Norwegian isolates in Clade A and in Japanese and Swiss isolates in Clade E and G. Fosfomycin has commonly been used in treatment of uncomplicated urinary tract infections but has now been reconsidered for the treatment of methicillin-resistant *S. aureus* (MRSA) infections. In Germany and Japan treatment with intravenous fosfomycin has been used successfully for four decades,^41,42^ and the resistance rates in German and Japanese isolates in our collection were 50.0% and 46%, respectively. It is worth noting that *fosB* is found only in isolates from the year 2000 onwards, possibly indicating a recent increase in resistance.

Plasmids encoding resistance to quaternary ammonium compounds (*qacA*, *qacC* and *qacG*), used in disinfectants,^43^ were widely distributed in all clades.

Fluoroquinolone resistance was caused by point mutations generating substitutions Ser80Phe in topoisomerase IV (GrlA) and Ser84Leu in gyrase A (GyrA). A total of 75/84 of the isolates dating from 2000 onwards, mainly found in Clades A, B, C and F, showed fluoroquinolone resistance. In contrast, isolates in Clade D, mainly isolated from 1990 to 2000, did not harbour these mutations. The observed fluoroquinolone resistance in isolates dating from 2000 onwards correlates well with the introduction and increase in usage of fluoroquinolones in European hospitals in the 1990s.^44^ In *S. aureus* fluoroquinolone resistance is associated with the emergence of MRSA and the successful *S. aureus* pandemic EMRSA-15 clone.^28^ Fluoroquinolones are commonly used to treat infections other than staphylococcal infections, so resistance might be beneficial for survival on human skin as fluoroquinolones are secreted in sweat and might affect skin commensals such as staphylococci.^28^ It is likely that the widespread use of antimicrobial agents has promoted the development of resistant clones of *S. haemolyticus* persisting in the hospital environment, which have responded by the acquisition of MGEs or beneficial core mutations. The uptake and loss of MGEs reflects the continuous and dynamic local adaption protecting the staphylococci from antimicrobial agents circulating in the hospital environment, thus enabling rapid adaption to changes in selective pressure.^45^

### SCCmec and ACME

The majority of the isolates (107/126; 84.9%) carried SCC*mec* (Table 1), encoding methicillin resistance by the production of alternative penicillin-binding protein, Pbp2a.^46^ In 62 of the *mecA*-positive isolates, mainly isolates belonging to Clades A and C, the regulatory gene *mecR1* was absent. The composition of the *mec* class was otherwise identical to SCC*mec* class C.^47^ *S. haemolyticus* isolates lacking the regulatory genes have previously been reported, albeit to a lesser extent.^48^ The absence of *mecR1* has been demonstrated not to interfere with the expression of *mecA*. This correlates well with phenotypic data showing that methicillin resistance was expressed in isolates lacking *mecRI*. Twenty-eight isolates had the previously described *mec* gene complexes C and A.^47^ *ccr* complex AB4 (31.7%) followed by *ccrC* (22.2%) were the most prevalent recombinases in our collection. Seventeen of the *mecA*-positive isolates harboured more than one type of recombinase, whereas 15 isolates harboured *mecA* but lacked *ccr* recombinase. Fifteen of the *mecA*-negative isolates harboured at least one set of *ccr* genes. A variation in the combination of recombinases with the *mecA* element has previously been reported for *S. haemolyticus*, indicating that it plays an important role in adaption.^49^

The ACME is a genetic island integrated in *orfX* next to SCC*mec*.^50^ The *arc* and/or *opp3* gene clusters encode one complete deaminase pathway and one oligopeptide permease system, respectively.^50^ ACME *arcA* was not detected in our *S. haemolyticus* collection. The novel allotypes *ccrA*-_SHP_ and *ccrB*-_SHP_ previously described in association with ACME *arcA* in *S. haemolyticus* were detected in 19 isolates.^51^ A low prevalence of ACME *arcA* in *S. haemolyticus* was previously reported by Onishi *et al*.,^52^ where ACME *arcA* was most prevalent in *S. epidermidis*.

Clustered regularly interspaced short palindromic repeats (CRISPRs) are known to limit the uptake of exogenous DNA.^53^ Only one isolate harboured what seemed to be a complete CRISPR system. This system had high sequence identity to the CRISPR genes found in *Staphylococcus lugdunensis* (GenBank accession number CP001837). The lack of functional CRISPR genes in most of the *S. haemolyticus* isolates examined could support the observed frequent uptake of MGEs.^54^

### Conclusions

We report for the first time the population structure of a large collection of clinical European *S. haemolyticus* isolates. Several multiply antibiotic-resistant European clones were detected, displaying the effects of antibiotic treatment on forming successful lineages. The presence of multinational clones with very recent separation in conserved clades may be an effect of human travel activity rather than a parallel evolution in different countries.

The majority of the collection was multiresistant, which corroborates previous observations of multiresistance in this species. The consequences of DNA acquisition and excision have been important in evolutionary terms since as many as one-fifth of the genes are part of the accessory genome and not shared by all the isolates. Recombination has also contributed significantly in shaping the genome as the SNP distribution pattern varies in different genomic regions, resulting in regional hotspots of recombination such as the *oriC* environs. The variation in the presence and level of recombination in surface-associated proteins and genes involved in pathogenicity, as well as bacterial communication in hospital-adapted isolates, indicate that these genes play an important role in the interaction between *S. haemolyticus* and the host. Both the frequent exchange of DNA and a high degree of recombination support the previous observation that *S. haemolyticus* has a highly flexible genome.^12^

## Funding

This work was supported by The Northern Norway Regional Health Authority. M. T. G. H., J. P. and S. D. B. were supported by Wellcome Trust grant ‘098051’. The Wellcome Trust Sanger Institute is core funded by Wellcome Trust grant number ‘098051’. S. D. B. is partly funded by the National Institute for Health Research (NIHR) Cambridge Biomedical Research Centre.

## Transparency declarations

None to declare.

## Supplementary data

Tables S1 and S2 are available as Supplementary data at *JAC* Online (http://jac.oxfordjournals.org/).

Supplementary Data
