# Supplementary material for: Whole-genome sequencing reveals clonal expansion of multiresistant Staphylococcus haemolyticus in European hospitals
Source: J Antimicrob Chemother. 2014 Jul 17;69(11):2920–7. doi: 10.1093/jac/dku271 (PMC4195474; doi:10.1093/jac/dku271)
Supplement: Supplementary Data [file supp_69_11_2920__index.html]

Whole-genome sequencing reveals clonal expansion of multiresistant Staphylococcus haemolyticus in European hospitals — Supplementary Data 

# Whole-genome sequencing reveals clonal expansion of multiresistant *Staphylococcus haemolyticus* in European hospitals

## Supplementary Data

Supplementary Data

**Files in this Data Supplement:**

- Supplementary Table 1 - xlsx file
- Supplementary Table 2 - docx file
