# Supplementary material for: Whole-genome sequencing reveals clonal expansion of multiresistant Staphylococcus haemolyticus in European hospitals
Source: J Antimicrob Chemother. 2014 Jul 17;69(11):2920–7. doi: 10.1093/jac/dku271 (PMC4195474; doi:10.1093/jac/dku271)
Supplement: Supplementary Data [file supp_dku271_dku271supp_table2.docx]

**Table S2. Distribution of methylases and restriction enzymes belonging to RM systems I-IV in clades A-F. Due to fragmented contigs the exact number of complete host restriction systems are not determined.**

|  | **No. isolates** | **Type I** | | **Type II** | | **Type III** | | **Type IV** | |
| --- | --- | --- | --- | --- | --- | --- | --- | --- | --- |
|  |  | **M^1^** | **R^2^** | **M** | **R** | **M** | **R** | **M** | **R** |
| Clade A | 39 | 2 | 5 | 165 | 30 | 41 | 39 | - | 97 |
| Clade B | 15 | 13 | 2 | 86 | 28 | 4 | 2 | - | 15 |
| Clade C | 17 |  | 3 | 179 | 23 | 22 | 14 | - | 22 |
| Clade D | 13 | 4 | 4 | 70 | 24 | 0 | 0 | - | 28 |
| Clade E | 7 | 4 | 9 | 30 | 14 | 0 | 0 | - | 12 |
| Clade F | 8 | 15 | 14 | 53 | 17 | 2 | 2 | - | 6 |
| Clade G | 23 | 17 | 26 | 34 | 33 | 9 | 5 | - | 20 |

^1^ Methylases, ^2^ Restriction enzymes
